# Editorial: New mechanisms for anti-cancer drugs

**DOI:** 10.3389/fphar.2024.1387942

**Published:** 2024-04-05

**Authors:** Ayaz Shahid, Ajit Prakash, Saad Mustafa, Pranav Kumar Prabhakar

**Affiliations:** ^1^ Department of Biotechnology and Pharmaceutical Sciences, College of Pharmacy, Western University of Health Sciences, Pomona, CA, United States; ^2^ Department of Biochemistry and Biophysics, School of Medicine, University of North Carolina at Chapel Hill, Chapel Hill, NC, United States; ^3^ Department of Geriatric Medicine, All India Institute of Medical Science, New Delhi, India; ^4^ Department of Research Impact and Outcome, Division of Research and Development, Lovely Professional University, Phagwara, India

**Keywords:** anti-cancer drug, nanotechnology-based drug delivery systems, natural product, biomarker, cell signaling

Cancer is one of the leading causes of death globally (Siegel et al., 2023). Ongoing investigations aim to discover and develop new drugs for treating cancer (Debela et al., 2021). However, identifying and evaluating the effectiveness of drugs poses a significant challenge. The Research Topic of “New Mechanisms for Anti-Cancer Drugs” is focused on gathering different studies that highlight innovative chemical or natural compounds with unique modes of action to target cell signaling pathways and exert cytotoxic effects on cancer cells. This Research Topic is an amalgamation of preclinical and clinical studies and review articles.

Overall, the studies presented in this Research Topic are significant in advancing our knowledge of cancer biology, biomarkers, and possible therapeutic approaches. The use of natural compounds, nanotechnology, and repurposed drugs represents the multifaceted efforts made to combat cancer. Together, these studies represent a promising step forward in the fight against cancer.

In a groundbreaking series of studies, investigators have discovered new insights into the potential of various compounds and plant extracts to combat different types of cancer. A study by (Petronek et al.) discovered that sirtinol, an intracellular iron chelator, can cause metabolic changes in non-small cell lung cancer (NSCLC) cells. Sirtinol also reduces labile iron pools and triggers an adaptive response that increases iron uptake and decreases storage, particularly in KRAS/STK11/KEAP1 mutant cells. This study demonstrated that sirtinol exhibits cytotoxic effects on NSCLC cells, which is attributed to its iron-chelating function. However, these effects are context-dependent and vary based on the genetic background differences in iron regulation and dependency between KRAS/STK11/KEAP1 mutant and wild-type cells. In another study, (Reddy et al.) found that Terminalia chebula fruit extract (TCF) has anti-proliferative effects against MCF-7 breast cancer cells by inducing cell cycle arrest at the G2/M phase and inhibiting EGFR signaling pathways. TCF phytochemical saccharopine shows potential as an anti-cancer agent, but further studies are needed to evaluate its efficacy and safety. A study by (Chandrasekaran et al.) discovered that cumin seed extracts are highly effective against bone cancer cell line MG63, showing an LC50 of 86 μg/mL. Cumin seed extracts have potential anti-cancer and antimicrobial properties with key compounds identified as phthalic acid, propanal, and methyl esters. In another study, (Rong et al.) suggest that vitamin D3 may prevent colorectal cancer by targeting mainly 10 proteins. It regulates biological processes and affects signaling pathways that control cancer mechanisms. Molecular docking analysis suggests it can directly bind to the predicted 10 proteins. Vitamin D3 anti-cancer properties were validated through bioinformatics analysis and animal experiments. These findings offer new prevention and treatment strategies for colorectal cancer. Wu et al. conducted a study that found curcumin can inhibit colon cancer cell growth and movement. The authors identified 3,505 genes upregulated in colon cancer cells treated with curcumin, including 37 genes acting as tumor suppressors. Molecular docking analysis showed that curcumin may disrupt the ARHGEF12-RhoA complex, suppressing cancer cell invasion. Another study by (Han et al.) revealed that maltol has anti-cancer properties against melanoma cells. It reduces melanin production and hinders the growth of cancer cells by inducing a pause in their cell cycle. Maltol also boosts apoptosis or programmed cell death and has a synergistic anti-cancer effect when combined with chemotherapy drugs. Additionally, maltol suppresses PD-L1 expression in melanoma cells, which can decrease immunotherapy resistance and enhance tumor cell killing. A study by (Chen et al.) aimed to investigate the influence of Danshen, a Chinese herbal medicine, on the clinical outcomes of bladder cancer patients. The study gathered data from Taiwan’s National Health Insurance database. Patients treated with Danshen had a lower incidence of major adverse cardiac events and improved survival rates. It was linked to a 44% reduction in MACE risk and a 40% decrease in mortality risk compared to non-Danshen patients, suggesting its potential to lower cardiovascular risks and improve outcomes. Together, these studies offer a multifaceted approach to cancer treatment and prevention, showcasing the diverse therapeutic potential of natural compounds in combating various cancer types.

An investigation led by Zhang and colleagues (Zhang et al.) has discovered that GLS, an overexpressed protein in multiple types of cancer, including breast cancer, can differentiate breast cancer cells from normal tissue samples. The reduction of GLS protein promotes the growth and spread of breast cancer cells, and its expression is associated with the immune system’s response to breast cancer. Therefore, GLS may serve as a useful diagnostic biomarker with potential functional implications for the diagnosis and treatment of breast cancer. Expanding on this theme, (Zhang et al.) identified 14 genes as prognostic markers for lung adenocarcinoma (LUAD) that are related to the basement membrane (BM). These genes are part of pathways that contribute to cancer progression. The study also established a link between the BM gene signature and immune cell infiltration. The authors developed a risk model based on these genes with good predictive ability. Additionally, the study identified 12 small-molecule drugs that have inhibitory effects against LUAD and suggested that cyclopamine and docetaxel could be more beneficial for low-risk patients. At the same time, Jamal and colleagues (Jamal et al.) found that the chemokine receptor CCR9 promotes the infiltration of T-ALL cells into tissues, leading to increased cholesterol biosynthesis and disease progression. It showed that statins could be beneficial in treating aggressive forms of breast cancer. To sum up, GLS is a diagnostic biomarker for breast cancer, while genes linked to the basement membrane are prognostic markers for lung adenocarcinoma. CCR9 promotes T-ALL cell infiltration, and statins may be used for treatment. These findings can enhance the accuracy of diagnosis and lead to personalized treatment options. In conclusion, these studies highlight the significance of ongoing research and collaboration in the battle against cancer.

Various studies also have shown that nanotechnology-based drug delivery systems can be effective in targeted cancer therapy, and these systems may have the potential to be further developed in the future. One such study by (Hussein et al.) aimed to determine the effectiveness of two types of niosomes in fighting cancer in breast cancer cell lines. The two types of niosomes tested were carnosine-loaded niosomes (Car-NIO) and melittin-loaded niosomes (Mel-NIO). The study showed that Mel-NIO has a stronger anti-cancer activity against breast cancer cells than Car-NIO. However, both Car-NIO and Mel-NIO are able to reduce lipid peroxidation and stop the progression of the cell cycle in cancer cells. In a similar vein, (Akbar et al.) developed nanoparticles of a metal-organic framework (MOF) coated with chitosan that is conjugated with folic acid (FC). The MOFs are bi-metallic FeCo-based MOFs (bi-MIL-88B), which selectively deliver an anti-cancer drug, 5-fluorouracil (5-FU), to FR-overexpressing cancer cells. The FC coating allows for pH-responsive and sustained release of 5-FU in the tumor microenvironment. This system has the potential for combined chemo and hemodynamic therapy. In conclusion, nanotechnology-based drug delivery systems have shown promising results in targeted cancer therapy. Both Car-NIO and Mel-NIO were able to reduce lipid peroxidation and stop the progression of the cell cycle in cancer cells. However, Mel-NIO was found to have stronger anti-cancer activity against breast cancer cells compared to Car-NIO. Akbar et al. developed MOFs coated with chitosan and conjugated with folic acid, which have the potential for combined chemo and hemodynamic therapy. These findings provide hope for developing more effective cancer treatments in the future.

Several review articles have highlighted the potential of natural products, such as flavonoids, alkaloids, and phytochemicals, in treating different cancers. A review article by (Khan et al., 2023) discussed the potential of plant-based compounds in reversing abnormal epigenetic changes in cancer cells. These plant-based compounds can alter DNA methylation, histone modifications, and microRNA expression. For example, compounds such as curcumin, EGCG, genistein, quercetin, and resveratrol can regulate the expression of genes involved in apoptosis, cell cycle control, DNA repair, etc., which are often dysregulated in cancer. However, their bioavailability and clinical evidence are still limited. Another review by (Zhou et al.) emphasized the potential of natural products such as flavonoids and alkaloids in treating endometrial cancer. These natural products have anti-cancer effects on endometrial cancer cells by regulating apoptosis. They act on signaling pathways that play a role in apoptosis, including the mitochondrial pathway, MAPK pathway, PI3K/AKT/mTOR pathway, and NF-κB pathway. Combining natural products with chemotherapeutic agents may have synergistic effects, but developing natural product-based therapies faces challenges of toxicity and bioavailability. (Zhan et al.) highlighted the numerous phytochemicals present in medicinal plants that can impede glycolysis and the Warburg effect in colorectal cancer cells. These phytochemicals target various enzymes, transporters, and pathways to affect glycolysis. Inhibition of the glycolytic pathway reduces proliferation and metastasis and increases sensitivity to chemotherapeutics in colorectal cancer. Furthermore, (Zhang et al.) discussed the potential of natural products to inhibit tumor growth and enhance cell death. These products target the endoplasmic reticulum stress (ERS) pathway, which promotes apoptosis in tumor cells. The authors summarized 69 natural products that can regulate ERS and induce apoptosis in various cancers. However, more research is needed to fully understand mechanisms, conduct clinical trials, and evaluate safety. In another review article by (Cao et al.), the alkaloid leonurine demonstrated anti-tumor properties in preclinical studies. It has been shown to inhibit cancer cell proliferation, induce apoptosis and autophagy, and reduce invasion and migration. However, further clinical research is necessary to validate the safety, pharmacology, and potential of leonurine as an anti-cancer agent or drug. In conclusion, plant-based compounds and natural products can target various aspects of cancer biology. However, challenges like bioavailability, toxicity, and clinical evidence persist, necessitating further research. These findings offer promising avenues for developing novel anti-cancer strategies based on natural products.

One study by (Wang et al.) found that abnormal lipid metabolism is a significant characteristic of cancer, especially hepatocellular carcinoma (HCC). Using statins, such as simvastatin, can reduce the risk of HCC and improve outcomes with immunotherapy in cancer patients. Combining statins with ICIs may improve immunotherapy in HCC, but determining the optimal dose and managing side effects remains challenging. Natural compounds and repurposed drugs are being explored as potential therapeutic strategies, but further research is needed to implement these approaches. Another review by (Zhang and Yu) explores the interaction between Wnt signaling and DNA damage response (DDR) pathways. These pathways, which comprise several components, including β-catenin, APC, Axin, GSK-3β, and p53, are crucial factors impacting cancer development and its resistance to therapy. Targeting the Wnt and DDR pathways simultaneously could help overcome therapy resistance in cancer. However, more research is needed to understand the molecular mechanisms involved in their interaction fully. Riccardi et al. explain how Antibody-drug conjugates (ADCs) work to deliver chemotherapy directly to cancer cells, reducing the damage to healthy cells. ADCs consist of a monoclonal antibody, a stable linker, and a cytotoxic payload. Their mechanism involves binding specific antigens on the surface of cancer cells, which triggers internalization and drug release. There are approved ADCs for cancer treatment, but they have toxicities related to cytotoxic payloads. Combination therapies and further research can expand their use. Buczyńska et al. highlight the therapeutic landscape beyond traditional boundaries, showing that medications used for treating diabetes, such as DPP-IV and SGLT2 inhibitors, can potentially be effective in treating papillary thyroid cancer (PTC). These drugs target glucose metabolism and the Warburg effect, which may help reduce PTC tumor growth and cell migration. However, further research is required to identify biomarkers for patient stratification and carry out well-designed clinical trials to evaluate the safety and efficacy of these drugs. Emerging therapies like Wnt/DDR inhibitors and natural products like leonurine hold potential, but more research is needed to realize their clinical promise.

This editorial concludes that various chemical compounds such as sirtinol, TCF, cumin seed extract, and Vitamin D3 have potential anti-cancer properties. It also discussed the anti-cancer effect of carnosine, melittin-loaded niosomes, and folic acid-coated metal-organic framework nanoparticles. Furthermore, it explains how GLS can be used as a diagnostic biomarker for diagnosing and treating breast cancer. Several review articles have also discussed prevention and treatment strategies for different types of cancers. All these findings bring hope for the future of cancer treatment as various anti-cancer compounds with unique mechanisms of action have shown potential for future cancer treatments.
